# Task and stimulus coding in the multiple-demand network

**DOI:** 10.1093/cercor/bhae278

**Published:** 2024-07-13

**Authors:** Sneha Shashidhara, Moataz Assem, Matthew F Glasser, John Duncan

**Affiliations:** Center for Social and Behaviour Change, Ashoka University, Sonipat, 131029, India; MRC Cognition and Brain Sciences Unit, School of Clinical Medicine, University of Cambridge, Cambridge CB27EF, United Kingdom; MRC Cognition and Brain Sciences Unit, School of Clinical Medicine, University of Cambridge, Cambridge CB27EF, United Kingdom; Departments of Radiology and Neuroscience, Washington University in St. Louis, Saint Louis, MO 63110, United States; MRC Cognition and Brain Sciences Unit, School of Clinical Medicine, University of Cambridge, Cambridge CB27EF, United Kingdom

**Keywords:** multiple-demand network, RSA, faces and houses, mixed selectivity, task demand

## Abstract

In the human brain, a multiple-demand (MD) network plays a key role in cognitive control, with core components in lateral frontal, dorsomedial frontal and lateral parietal cortex, and multivariate activity patterns that discriminate the contents of many cognitive activities. In prefrontal cortex of the behaving monkey, different cognitive operations are associated with very different patterns of neural activity, while details of a particular stimulus are encoded as small variations on these basic patterns (Sigala et al, 2008). Here, using the advanced fMRI methods of the Human Connectome Project and their 360-region cortical parcellation, we searched for a similar result in MD activation patterns. In each parcel, we compared multivertex patterns for every combination of three tasks (working memory, task-switching, and stop-signal) and two stimulus classes (faces and buildings). Though both task and stimulus category were discriminated in every cortical parcel, the strength of discrimination varied strongly across parcels. The different cognitive operations of the three tasks were strongly discriminated in MD regions. Stimulus categories, in contrast, were most strongly discriminated in a large region of primary and higher visual cortex, and intriguingly, in both parietal and frontal lobe regions adjacent to core MD regions. In the monkey, frontal neurons show a strong pattern of nonlinear mixed selectivity, with activity reflecting specific conjunctions of task events. In our data, however, there was limited evidence for mixed selectivity; throughout the brain, discriminations of task and stimulus combined largely linearly, with a small nonlinear component. In MD regions, human fMRI data recapitulate some but not all aspects of electrophysiological data from nonhuman primates.

## Introduction

**Fig. 1:**
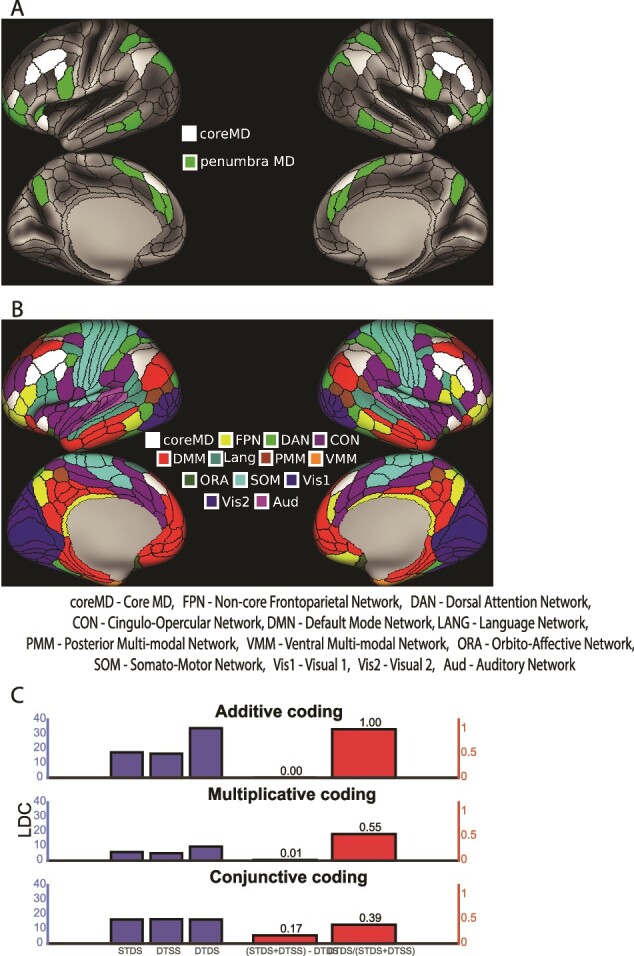
A) 10 core MD parcels (white) and 17 penumbra MD parcels (green) from Assem et al. (2020). B) 360 cortical parcels from Glasser et al. (2016), organized into the 13 resting-state networks identified in Ji et al (2019). Core MD parcels (Assem et al. 2020) are a subset of the FPN from Ji et al. (2019); here we show core MD in white, and remaining FPN parcels in yellow. C. Simulated results (see text) for brain regions with varying relationships between task and stimulus representations. Measure is LDC between activity patterns for same task different stimulus (STDS), different task same stimulus (DTSS), and different task different stimulus (DTDS). To measure nonlinearity (right), we use (STDS + DTSS) – DTDS (linked to the left y-axis) and DTDS / (STDS + DTSS), (linked to the right y-axis). Top: Additive, with activation in each voxel a sum of influences from task and stimulus. Middle: Multiplicative, with activation in each voxel multiplying task and stimulus influences. Bottom: Conjunctive, with unique activity level for each task-stimulus combination.

In the human brain, “multiple-demand” or MD regions are characterized by increased activity associated with many different kinds of cognitive demand (Duncan and Owen, 2000; Duncan et al. 2020). Over the past two decades, this MD system has been defined with increasing anatomical precision. A recent version from Assem et al. (2020) is shown in Fig. 1A. To produce this version, Assem et al. (2020) used data from 449 participants in the Human Connectome Project (HCP), relating results to the Glasser et al. (2016) parcellation of the human cerebral cortex into 180 regions per hemisphere. Overlapping activity for three types of cognitive demand was strongest in a set of 10 regions/hemisphere, distributed over the lateral frontal, dorsomedial frontal, insular, and lateral parietal cortex (Fig. 1A, white). Assem et al. (2020) called this set of 10 regions the MD core, with additional, weaker shared activity across all three contrasts in an accompanying penumbra of 17 further regions (Fig. 1A, green). HCP resting-state data showed strong functional connectivity between the 10 core regions, despite their wide distribution across the cortex. Cross-reference to a previous definition of resting-state networks in this 180-region cortex (Fig. 1B; Ji et al. 2019) showed that the 10 core MD regions are a subset of the well-known “frontoparietal control” network (FPN), with penumbra regions distributed across both additional FPN regions and several other networks.

For each cognitive operation, multiple components must be assembled into a computational structure capturing their required roles and relationships. The MD core is well placed to effect such “attentional integration” (Duncan et al. 2020), with parts widespread throughout the cortex, affording strong local access to many kinds of cognitive content, and strong functional connectivity, affording communication and integration. Indeed, a recent study of three executive functions—working memory (WM), switching and inhibition—shows differential activation of several regions immediately adjacent to the MD core, and within the core, partially overlapping activations, but each biased towards these different adjacent regions (Assem et al. 2024). Similarly, using visual and auditory WM tasks, (Assem et al. 2022) showed overlapping activity in the MD core, but just ventral to these core regions, alternating patches of visual and auditory preference on the lateral frontal surface (see also Michalka et al. 2015; Noyce et al. 2017; Tobyne et al. 2017).

As required for a system that represents task-relevant content, multivariate pattern analysis shows much encoding of task-relevant stimuli, rules, and responses in MD regions (Woolgar et al. 2011; 2015; 2016; Crittenden et al. 2015; Erez and Duncan, 2015; Cole et al. 2016; Etzel et al. 2016; Smith et al. 2018; Shashidhara et al. 2020; Shashidhara and Erez, 2021). Such results are reminiscent of highly adaptable responses in single neurons of the monkey frontal and parietal cortex, with large fractions of neurons showing selective responses to particular task events and shifts in response properties to track the most relevant information in any one task context (Duncan, 2001; Freedman et al. 2001; Meyers et al. 2008; Sigala et al. 2008; Duncan and Miller, 2009; Swaminathan and Freedman, 2012; Rigotti et al. 2013; Stokes et al. 2013). Here we re-analyse the data from Assem et al. (2024) to address two questions prompted by these single neuron data.

One common property of frontal and parietal neurons is discrimination of relevant stimuli and stimulus categories (Freedman et al. 2001; Freedman and Assad, 2006; Meyers et al. 2018). Much stronger, however, may be coding of different cognitive operations applied to these stimuli. In an early study, Wallis et al. (2001) presented pairs of stimuli, cueing on each trial whether response should be made when the two were similar or when they were different. Though some frontal neurons discriminated individual stimuli, many more distinguished the response rule. In a similar vein, (Sigala et al. 2008) examined patterns of frontal activity across successive stages of a complex task, each stage requiring a different cognitive operation. Activity patterns were almost orthogonal for different task stages, while stimulus discriminations within each stage were reflected in small modulations of these patterns (see also Kadohisa et al. 2020). Here, within every region of the Glasser at al. (2016) parcellation, we used multivariate pattern analysis to compared discrimination of cognitive operation—WM, switching, or inhibition—with discrimination between two very distinct stimulus categories used in the study, faces and buildings. With multivariate methods, we expected to capture both the univariate task differences already reported by Assem et al. (2024), and any further discrimination captured by fine-scaled pattern differences. By grouping 180 parcels/hemisphere into the resting state networks defined by Ji et al. (2019), we compared core MD with the brain’s other major networks.

A second important property of the frontal single neuron data is mixed selectivity; very often, activity depends on the conjunction of multiple task features, such as a particular stimulus object presented at a particular place in a memory list, or a particular move planned at a certain position in a sequence (Mushiake et al. 2006; Sakamoto et al. 2008; Warden and Miller, 2010; Rigotti et al. 2013). Here, we asked whether selectivities for cognitive operation and stimulus class interact in a way suggestive of conjunctive coding. The simulated results in Fig. 1C illustrate the approach. Using a simulated cortical region of 100 vertices, we calculated similarities of multivertex patterns for two tasks and two stimulus classes, for purely additive, multiplicative and purely conjunctive patterns. For the additive model, each vertex was assigned four base values, one for task A, one for task B, one for stimulus class A, and one for class B, each randomly selected between 0 and 1. To calculate net activation in any one combination of task and stimulus, the two relevant values were added together. This would be analogous to a neuron with independent selectivities for task and stimulus. After the addition of Gaussian noise to data from each simulated run of data collection, we measured similarities between the four 100-vertex patterns using linear discriminant contrast (LDC; see Methods) and averaged results over 1,000 such simulations. We define STDS as the LDC distance between patterns for same task different stimulus, DTSS as mean distance between patterns for different task same stimulus, and DTDS as mean distance between patterns for different task different stimulus. As expected (Fig. 1C, top row), the effects of different task and different stimulus sum linearly. As one measure of interaction (right), we subtract DTDS from the sum of DTSS and STDS. A second measure is the ratio between DTDS and the sum of STDS and DTSS. For this linear model, the first index is zero, and the second is one. In the second row of Fig. 1C, we show results for a multiplicative model, with activation in each vertex given by multiplication of its values for task and stimulus. This is analogous to a neuron with especially strong response for one task + stimulus combination. For this model the first interaction score is slightly above zero, and the second substantially below one. In the third row we show results for a fully conjunctive model. For this model, we assigned independent, random activations for each conjunction of task and stimulus. This would be analogous to neurons with selective response to a unique task-stimulus conjunction. Now, necessarily, the three types of distance were the same, producing strong underadditivity between the effects of task and stimulus difference.

Evidently, fMRI responses could be underadditive for other reasons than conjunctive coding. For example, underadditivity would arise if activity tended to saturate, for either neural or hemodynamic reasons. Here, we compared patterns of additivity/interaction across brain networks, asking whether strong underadditivity was seen in MD regions.

## Methods

### Participants

A total of 50 healthy participants (23 female, mean age 25.9 years) took part in the study. Four participants were excluded because of movements larger than 5 mm during at least one of the scanning runs, and five more participants owing to incomplete data. In addition, two participants were excluded owing to technical problems during scanning. Lastly, two participants were excluded owing to problems during analysis. Overall, 37 participants were included in the analysis. All participants were right-handed and had normal or corrected-to-normal vision. Participants gave written informed consent before participation and received monetary compensation at the end of the experiment. Ethical approval was obtained from the Cambridge Psychology Research Ethics Committee.

### Experimental paradigm

The study consisted of three visual executive tasks that were intermixed within a scanning run. They were: n-back WM, task-switching, and stop-signal, variations of which have previously been shown to recruit the MD network (Fedorenko et al. 2013). Participants practiced all tasks before the start of the scanning. During scanning, participants performed four runs of 36 blocks each: 8 n-back, 8 task-switch, 8 stop-signal, and 12 fixation blocks. The eight blocks of each task consisted of four Hard and four Easy, two of each using faces and two buildings. Hard and Easy blocks were always run in adjacent pairs, the two in a pair using the same stimuli, and Hard and Easy separated by a fixation block. Orders of Hard/Easy, and of the three tasks and two stimulus types, were randomized in each run. Note that for present purposes only data from Hard blocks were analysed, as executive demands were either minimal or absent in Easy blocks (see below).

Each block started with a cue indicating the forthcoming task (4 s), followed by 12 trials of 2 s each, and an inter-block-interval of 2 s (blank screen). Each trial had a 1500 ms stimulus presentation followed by a 500 ms blank screen. Responses were accepted until the end of the trial (2 s). Thus, all task blocks lasted for 30 s, and fixation blocks for 16 s, leading to a total run duration of 15 min and 12 s. The total scanning session duration was around an hour.

Face stimuli were selected from the Developmental Emotional Faces Stimulus Set (Meuwissen et al. 2017). Faces differed across three dimensions: they were either males or females, children or adults, with a happy or sad expression. Building stimuli were houses or churches, old or modern, shown from the inside or outside. There were 32 faces and 32 buildings, with each of the 2 × 2 × 2 possible feature combinations having four exemplars each. The image displayed on the screen covered 452 × 454 pixels and spanned 6 deg visual angle in height and 8 deg in width.

All tasks were coded and presented using Psychtoolbox (Brainard, 1997) for MATLAB (The MathWorks, Inc.). Stimuli were projected on a 1920 × 1080 screen inside the scanner, and participants used two button boxes to respond. Many participants used two fingers of the right hand as instructed, but some used index fingers of both hands.

### Tasks

#### Task 1: n-back WM

**Fig. 2:**
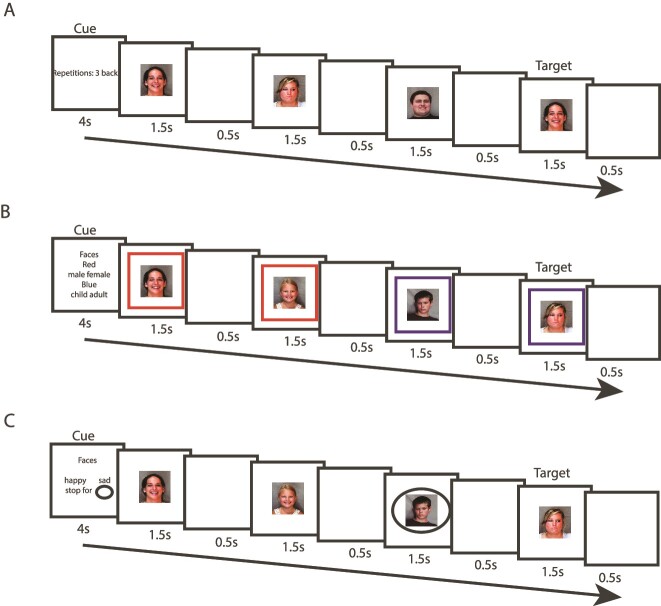
Schematic overview of the Hard condition of the three tasks. A) n-back. Participants were presented with blocks of faces and buildings (not shown). The task was to detect occasional targets (repetition of the image three steps back in the sequence). B) Task-switching. Participants made a judgement on each stimulus based on the cued rule. A red border indicated the male v. female rule for faces and a house v. church distinction for buildings (not shown), and a blue border indicated a child v. adult distinction for faces and an indoor v. outdoor distinction for buildings (not shown). C) Stop-signal. Participants made a happy v. sad judgement on faces (old v. modern on buildings, not shown) but refrained from pressing when a black oval appeared.

On each trial, a single face or building was presented at the center of the screen (Fig. 2). The task was to detect occasional targets, defined as a match to the immediately preceding stimulus (Easy, 1-back) or to the stimulus three places back in the sequence (Hard, 3-back). Using a keypad, participants pressed right for targets and left for nontargets. In half of the blocks there was a single target, in the others two targets.

#### Task 2: Task-switching

In task-switch blocks, each central face or building was surrounded by a square frame, either red or blue (Fig. 2B). For face stimuli, the red border indicated a gender rule, where participants responded left for male and right for female. The blue border indicated an age rule, left for a child and right for an adult. For building stimuli, the red border indicated a house or church rule, left for house and right for church, while the blue border indicated indoor/outdoor, indoor left and outdoor right. In Easy blocks frame color was fixed, with an equal number of red and blue blocks, while in Hard blocks, frame color varied pseudo randomly, ensuring an equal number of trials with each rule.

#### Task 3: Stop-signal

On each trial, there was a single face or building at screen center (Fig. 2C). For faces, participants pressed left for happy, right for sad. In building blocks, they pressed left for old and right for modern. Additionally, they were asked to withhold pressing a button if they saw a black circle around the stimulus. This stop-signal appeared only in Hard blocks and appeared 100 ms before the average RT for that participant on all go trials of the same stimulus category, across all previous trials including the practice session. Each Hard block had four stop trials.

### fMRI data acquisition

Participants were scanned in a Siemens 3T Prisma MRI scanner with a 32-channel head coil. MRI CCF acquisition protocols for the HCP Young Adult cohort were used (package date 2016.07.14; https://protocols.humanconnectome.org/CCF/). T1-weighted 3D multiecho MPRAGE (van der Kouwe et al. 2008) and 3D T2w SPACE structural images (voxel size 0.8 mm isotropic) were obtained. Rest fMRI (2 runs × 15 min) was acquired in a separate session. Task and rest fMRI were acquired using identical EPI sequence parameters (multiband = factor 8, voxel size of 2-mm isotropic resolution, TR = 800 ms, TE = 37 ms). EPI runs were acquired in pairs of reversed phase-encoding directions (AP/PA). Spin echo phase reversed images (AP/PA) were acquired during the structural and functional (after every two functional runs) scanning sessions to correct T1w and T2w images for readout distortion.

### Data Analysis

#### Pre-processing

Our data preprocessing followed the steps of HCP’s minimal preprocessing pipelines (Glasser et al. 2013), using HCP pipelines version 3.27.0 (scripts available at: https://github.com/Washington-University/HCPpipelines). Structural images of T1w and T2w were used to extract cortical surfaces and segmentation of subcortical structures, separately for every subject. Functional images (rest and task) were mapped from volume to surface space and combined with subcortical data in volume and smoothed by a 2 mm FWHM kernel in the standard CIFTI gray ordinates space. This approach avoids mixing data across gyral banks for surface data and avoids mixing areal borders for subcortical data.

HCP pipelines version 4.0.0 were used to additionally clean up spatially specific noise using spatial ICA + FIX in rest and task fMRI data (Salimi-Khorshidi et al. 2014). ICA+FIX was applied separately to all concatenated rest runs and all visual runs. An improved FIX classifier was used for more accurate classification of noise components in task fMRI datasets and manual checking of ICA + FIX outputs for 10 subjects led to fixing a threshold of 50 for “good” vs “bad” signal classification, then applied for the remaining subjects. Multimodal surface matching algorithm MSM was used for accurate registration of cortical surfaces. First “Sulc” cortical folding maps are gently registered in the MSMSulc registration, optimizing for functional alignment without overfitting folds. Second, a combination of myelin, resting-state network, and rest fMRI visuotopic maps (Robinson et al. 2014, 2018) was used to fully functionally align the data. For this purpose, we collected 30 mins resting state data in a second session, not discussed here.

#### General linear model (GLM)

Autocorrelation was estimated using FSL’s FILM on the surface and default parameters in HCP’s task fMRI analysis scripts. A GLM was estimated for each participant. For each task, regressors were created for Easy and Hard blocks, separately for each of the two stimulus classes. These were block-wise regressors from cue onset to the last trial (28 s), convolved with a canonical hemodynamic response function (HRF) and its temporal derivative. GLM used twelve movement parameters as covariates of no interest. Data and GLM model were temporally filtered with a Gaussian-weighted linear high-pass filter with a cutoff of 200 s and the time series was prewhitened within FILM to correct for autocorrelations in the fMRI data. FSL's FILM used a surface-based autocorrelation smoothing estimate at a sigma of 5 mm. The resulting β-estimates were used to generate beta “cope” maps using custom-built MATLAB scripts. Percent signal change was computed as follows: 100*(beta/10,000), where 10,000 corresponds to the mean scaling of each vertex time series during preprocessing. Percentage signal values were then used for pattern analyses. For parcellating the cerebral cortex, the group-average HCP multimodal parcellation (MMP1.0) was used (Glasser et al. 2016).

#### Pattern analysis using LDC

For each participant, we obtained the multivoxel pattern of activation in each of the 360 cortical parcels. To measure dissimilarity between activation patterns, we used LDC (Nili et al. 2014; Carlin and Kriegeskorte, 2017). Patterns were obtained for each of the six task + stimulus conditions, and cross-validated Mahalanobis distances were calculated for all 15 across-condition pair wise combinations. For each pair of conditions, we used one run as the training set and another run as the testing set. This was done for all pairwise combinations of the four runs and LDC values were then averaged across them. Larger LDC values indicate more distinct patterns of the tested conditions. The choice of using LDC rather than LD-t (associated t-value) meant that we could meaningfully look at differences between distances. To better compare distances across parcels, we divided LDC value for the whole parcel by the total number of vertices it contained. The number of vertices varied across the 360 parcels. The average number was 165.03 (range: 31–839). The average of the 20 core MD parcels was 188.45 (range: 63–509).

#### Task and stimulus distances

For each parcel, from the full matrix of LDC distances we calculated three means. STDS was calculated as mean distance between face and building blocks in the same task, averaged across tasks. DTSS was calculated as mean distance between tasks holding stimulus category constant, e.g. n-back face and task switch face, averaged across all task pairs and across face and building blocks. DTDS was calculated as mean distance between conditions differing in both task and stimulus category, averaged across six such distances. The nonlinearity index was calculated as (STDS + DTSS) − DTDS, and DTDS / (STDS + DTSS) (Fig. 1C).

#### Ventricle extraction

As a control analysis of discrimination where no true signal is possible, we examined data from the brain ventricles. This analysis was conducted in MNI space. The ventricles were segmented for each subject separately using FreeSurfer’s standard segmentation. We selected the left and right lateral ventricular masks. As part of the HCP preprocessing pipeline, the masks were transformed to MNI space. Masks were further eroded by 2 mm to avoid bleed of signals from nearby subcortical structures or white matter. All cleaning steps applied to the surface data were also applied automatically to the volumetric data (i.e. identical cleaning with ICAFIX). Unlike surface analysis, no smoothing was performed. For the resulting ventricular ROI, task and stimuli distances were analysed just as they were for cortical parcels.

#### Statistical testing and code

We used an alpha level of .05 for all statistical tests. Bonferroni correction for multiple comparisons was used when required, and the corrected *P*-values and uncorrected t-values were reported. All analyses were conducted using custom-made MATLAB (The MathWorks, Inc.) scripts unless otherwise stated.

### Data and code availability statement

Anonymized data and code will be available in a public repository before publication. Data and code sharing are per institutional procedures and ethics approval.

## Results

### Behavioral results

The mean accuracies and reaction times (RTs) for all trials in the Hard conditions of n-back, task-switching, and stop-signal tasks are listed in Table 1. A two-way repeated measures ANOVA of RTs with task Assem et al. 2024 and stimulus category Assem et al. 2022 showed a task effect (*F_2_* = 18.14, *P* < 0.001), a category effect (*F_1_* = 59.07, *P <* 0.001) with face trials being faster, and an interaction between the two (*F_2_* = 11.47, *P <* 0.001). Overall, n-back was faster than both task-switching and stop-signal (*t_36_* > 4.21, *P <* 0.001), and there was no difference between task-switching and stop-signal. There was a category effect with faces being faster in both task-switching and stop-signal (*t_36_* > 4.27, *P <* 0.001), but not in n-back.

**Table 1:** RTs and accuracies in each condition. Values are means ± standard errors.

	Hard n-back		Hard task-switching		Hard stop-signal	
	Face	Building	Face	Building	Face	Building
RT (s)	0.79 ± 0.02	0.80 ± 0.02	0.95 ± 0.01	1.03 ± 0.02	0.96 ± 0.04	1.01 ± 0.04
Accuracy (% correct)	88.89 ± 1.12	86.55 ± 1.11	92.86 ± 0.88	91.23 ± 0.80	70.86 ± 1.07	71.66 ± 1.08

A two-way repeated measures ANOVA of accuracies with task Assem et al. 2024 and stimulus category Assem et al. 2022 showed a task effect (*F_2_* = 181.72, *P <* 0.001), with no category effect or interaction. Task-switching was more accurate than both n-back and stop-signal (*t_36_* > 3.96, *P <* 0.001), and n-back was more accurate than stop-signal (*t_36_* = 12.46, *P <* 0.001).

Separating trials by target and nontarget in N-back showed faster RTs (only correct trials) and greater accuracy for nontarget trials in trials (RT nontarget: 0.75 ± 0.12 s, target: 0.82 ± 0.11 sec; *t_36_* = 4.20, *P <* 0.001; accuracy nontarget: 96.83 ± 1.59 %, target: 78.07 ± 10.21 %, *t_36_* = 11.67, *P <* 0.001). Task-switching showed no difference in RT (only correct trials) or accuracy between switch and stay trials (RT stay: 0.99 ± 0.09 s, switch: 0.98 ± 0.09 s; *t_36_* = 0.33, *P* = 0.75; accuracy nontarget: 91.80 ± 5.01 %, target: 92.31 ± 4.76 %, *t_36_* = 0.74, *P =* 0.47). In the stop-signal task, as correct stop trials do not have a reaction time, we report only accuracy. The go trials have higher accuracy compared to the no-go/stop trials (RT go: 1.06 ± 0.27 s; accuracy go: 95.73 ± 3.6 %, stop: 46.79 ± 10.17 %, *t_36_* = 27.17, *P <* 0.001).

### Task and stimulus discrimination across parcels

**Fig. 3:**
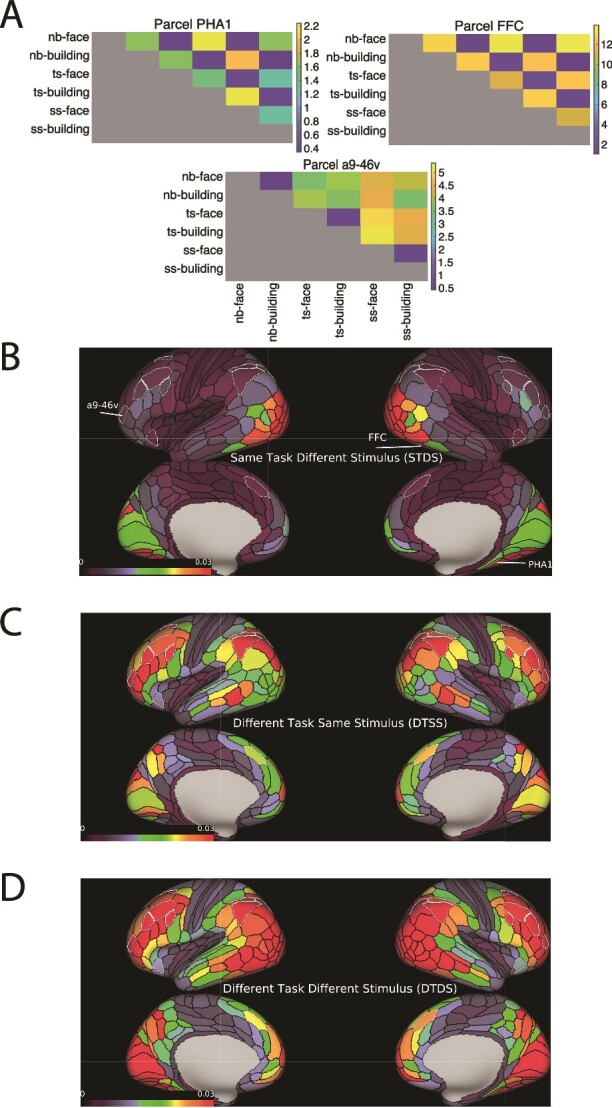
A) Sample RDMs (Representational Dissimilarity Matrix) of three cortical parcels. The matrices show the dissimilarities (LDC values) between all pairs of conditions. The diagonal is defined as zero. PHA1 (building area) and parcel FFC (face area) show strong stimulus discrimination, with similar activation patterns for the same stimulus category in different tasks and dissimilar patterns for faces and buildings. Parcel a9-46v (core MD) shows a complementary result, with similar activation patterns for faces and buildings in the same task. nb, ts, and ss refer to n-back, task-switching and stop-signal tasks respectively. Face and building refer to blocks where the stimuli were faces or buildings. B, C, and D) Mean LDC distance in each parcel for differences in stimulus (STDS), task (DTSS) and both (DTDS). White borders indicate core MD parcels.

Across the 360 parcels of the cortex, some showed strong discrimination of stimulus category, others of task. Examples are shown in Fig. 3A. Parcel PHA1 (building area in higher ventral visual region) and FFC (face area) showed strong stimulus discrimination (Fig. 3A), with similar patterns for the same stimuli in different tasks, and dissimilar patterns for faces and buildings. Parcel a9-46v, part of the core MD network (Assem et al. 2020), showed a complementary result, with similar activation patterns for the two stimulus categories in each task.

For each parcel we calculated our three discrimination indices, STDS, DTSS, and DTDS (see Methods). Results are shown in Figs. 3B–D. Unexpectedly, all three indices were significantly greater than zero in every parcel in the brain, Bonferroni corrected for 360 comparisons (*t_36_* > 6.44, *P <* 0.001). Randomly sampling subjects 10,000 times with replacement gave a significant STDS in all parcels more than 99.83% of times, and a significant DTSS and DTDS 100% of times. Against this background, however, discriminations of stimulus and task showed very different cortical distributions.

For STDS, as expected, discrimination was strongest in a large region of early and higher visual cortex (Fig. 3B, red to green). Weak discrimination in core MD regions was accompanied by somewhat stronger discrimination close by: in lateral parietal cortex, in a band of regions ventral to MD core; in lateral frontal cortex, in a similar band of regions also ventral to MD core; and in dorsomedial frontal cortex, in a region anterior and ventral to MD core. In posterior temporal and medial parietal cortex, similar levels of discrimination extended towards and into MD penumbra (compare Figs. 1A and 3B).

For DTSS, in contrast, discrimination was strong in MD core and adjacent regions, many included in MD penumbra (Fig. 3B). Discrimination was also strong in and adjacent to penumbra regions of posterior temporal and medial parietal cortex.

For DTDS, as expected, discrimination was widespread, reflecting the union of patterns for STDS and DTSS.

Finally, as a sanity check for significant discrimination in all parcels, we calculated the same three indices for data from a ventricle ROI (see Methods). As expected, none of the three indices was significant in this ROI (*t_36_* < 1.47, *P* > 0.13).

### Network distances

**Fig. 4:**
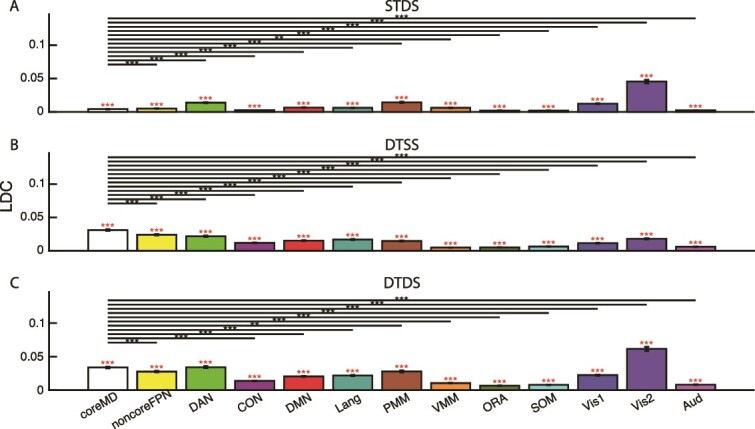
Mean values of STDS (A), DTSS (B), and DTDS (C). Red asterisks above each bar show significant difference from 0 (Bonferroni corrected for 13 comparisons). Lines and black asterisks show significant differences between core MD and other networks (Bonferroni corrected for 12 comparisons).

To summarize these results, we used the 12 cortical networks defined by Ji et al. (2019), with their FPN divided into core MD and the remainder (Fig. 1B). Values of our three discrimination indices were averaged across parcels in each network, and across the two hemispheres. Results are shown in Fig. 4.

For STDS (Fig. 4A), a one-way repeated measures ANOVA showed a main effect of network (*F_12,432_* = 219.63, *P <* 0.001). Individual t*-*tests compared core MD with each of the other networks. All 12 comparisons were significant (*t_36_* < -4.06, *P <* 0.003, Bonferroni corrected for 12 comparisons), with STDS lower in core MD than in remaining noncoreFPN, DAN, DMN, Lang, PMM, VMM, Vis1, and Vis2, and higher in core MD than CON, SOM, ORA, and Aud. Separate t-tests showed that STDS was significantly greater than zero in all 13 networks (*t_36_* > 12.04, *P <* 0.001, Bonferroni corrected for 13 comparisons).

For DTSS, similarly, one-way repeated measures ANOVA showed a main effect of networks (*F_12,432_* = 268.76, *P <* 0.001). Core MD has higher DTSS than all networks (*t_36_* > 10.38, *P <* 0.001, Bonferroni corrected for 12 comparisons) (Fig. 4B). Separate t-tests showed that DTSS was significantly greater than zero in all 13 networks (*t_36_* > 15.56, *P <* 0.001, Bonferroni corrected for 13 comparisons).

For DTDS (Fig. 4C, one-way repeated measures ANOVA also showed a main effect of network (*F_12,432_* = 247.62, *P <* 0.001). DTDS was higher in core MD than in all other networks (*t_36_* > 4.15, *P <* 0.001, Bonferroni corrected for 12 comparisons), except Vis2 (*t_36_* = -11.14, *P <* 0.001, Bonferroni corrected for 12 comparisons) and DAN (*t_36_* = -0.39, *P >* 0.9, Bonferroni corrected for 12 comparisons). Separate t-tests showed that DTDS was significantly greater than zero in all 13 networks (*t_36_* > 17.86, *P <* 0.001, Bonferroni corrected for 13 comparisons).

### Nonlinearity

**Fig. 5:**
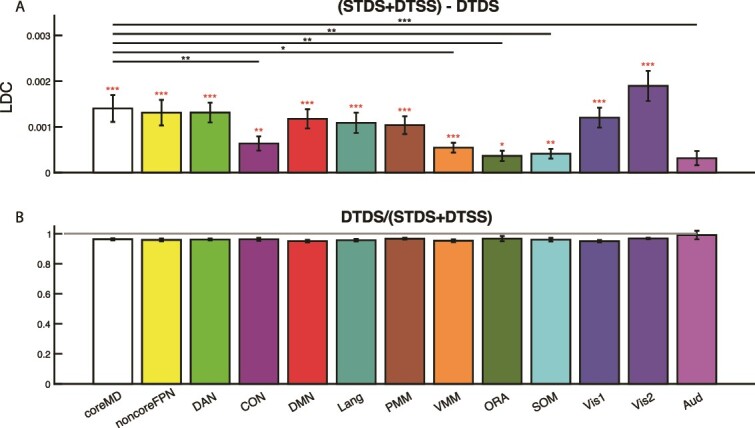
A) Nonlinearity index for all networks. Red asterisks above each bar show significant difference from 0 (Bonferroni corrected for 13 comparisons). Lines and black asterisks show significant differences between core MD and other networks (Bonferroni corrected for 12 comparisons). B) Values of the ratio DTDS / (STDS + DTSS). A ratio of one (grey line) would indicate complete linearity.

For each network, mean values of the nonlinearity index, (STDS + DTSS) – DTDS, are show in Fig. 5A. A one-way repeated measures ANOVA showed a main effect of network (*F_12,432_* = 15.94, *P <* 0.001). Individual t-tests showed that the index was positive in all networks (*t_36_* > 3.22, *P <* 0.04, Bonferroni corrected for 13 comparisons), except Aud. Again, we compared core MD with each other network. The nonlinearity index was higher in core MD than in CON, VMM, ORA, SOM, Aud (*t_36_* > 3.52, *P <* 0.05, Bonferroni corrected for 12 comparisons). Another way to visualize nonlinearity is the ratio of DTDS to the sum of STDS and DTSS (Fig. 5B). For this index, a value of one would indicate complete linearity. A one-way repeated measures ANOVA showed no main effect of network. The ratio was not different between any two networks (*t_36_* < 2.76, *P >* 0.11, Bonferroni corrected for 12 comparisons). Though the ratio was less than 1 in all networks (*t_36_* < -3.33, *P <* 0.03, Bonferroni corrected for 13 comparisons) except ORA and Aud, a minimum value of 0.95 (Vis1) shows close to perfect linearity in all networks.

To understand the limits of nonlinearity in the brain we looked at nonlinearity for the top 20 parcels that showed highest discrimination between tasks and stimuli. The parcels were ranked based on their task and stimulus discrimination, 1–360 where 1 is highest. These ranks were added and the index: DTDS / (STDS + DTSS) was computed for top 20 parcels (mean: 0.96 ± 0.01) with the highest rank and bottom 20 parcels (mean: 0.95 ± 0.02) with the lowest rank. A t-test between the two sets showed no difference (*t_36_* = 1.76, *P =* 0.095).

Finally, to compare the network values with the simulations in Fig. 1C, we created some hybrid simulations that included additive and conjunctive vertices. For additive vertices, using a fixed level of noise, we varied the magnitude of stimulus and task discriminations (range of selected values for each voxel) to approximately match levels seen in the experimental data for core MD. To a purely additive model, we added varying proportions of conjunctive voxels, with activation values independently selected for each stimulus-task combination (using a range intermediate between those for stimulus and task). We varied the proportion of additive vertices from 0 (Fig. 1C, bottom row) to 100 (Fig. 1C, top row). As expected, these simulations showed an increase in linearity with more additive vertices (Table 2). Values of STDS, DTSS, and DTDS all matched core MD data at a level of around 90% additive vertices. Similar results were obtained for match to a second network, PMM, with more similar effects of stimulus and task (Fig. 4). Many factors limit direct comparison between these simple simulations and the data. At the same time, the results provide one possible benchmark for assessing the degree of linearity in the experimental findings.

**Table 2:** Non-linearity indices for a mix of additive and conjunctive vertices.

Proportion of additive vertices out of 100	STDS	DTSS	DTDS	(STDS + DTSS) − DTDS	DTDS / (STDS + DTSS)
0	0.0157	0.0157	0.0157	0.0158	0.5
0.5	0.0094	0.024	0.0256	0.0079	0.7651
0.6	0.0082	0.0256	0.0275	0.0063	0.8137
0.7	0.007	0.0275	0.0297	0.0048	0.8615
0.8	0.0057	0.0291	0.0316	0.0032	0.9108
0.9	0.0045	0.0307	0.0336	0.0016	0.9563
0.91	0.0043	0.0309	0.0338	0.0014	0.9614
0.93	0.0041	0.0312	0.0342	0.0011	0.971
0.95	0.0038	0.0315	0.0345	0.0008	0.9787
0.97	0.0036	0.0319	0.035	0.0005	0.9883
0.99	0.0033	0.0322	0.0354	0.0002	0.9969
1	0.0032	0.0324	0.0356	0	1

## Discussion

In this study we used multivariate pattern analysis to examine discrimination of task and stimulus category in the MD system. As we had predicted from single cell activity in the lateral frontal cortex of the monkey, we found strong discrimination of our three tasks, requiring different executive operations. Discrimination was strong throughout MD core and extended to a number of additional regions in the MD penumbra. In contrast, core MD regions showed only weak discrimination of stimulus category. On lateral parietal, lateral frontal and dorsomedial frontal surfaces, somewhat stronger stimulus discrimination was seen in several regions adjacent to MD core, while in the temporal and medial parietal lobes, similar levels of discrimination extended into MD penumbra.

In our previous analyses of this data set (Assem et al. 2024), we used standard univariate analysis to show significant task differences in several regions adjacent to MD core. Especially in the right hemisphere, for example, the stop signal task showed strongest activation in regions belonging to the cingulo-opercular network (Fig. 1B), while especially in the left hemisphere, the task switch showed strongest activation in regions of the dorsal attention network. Within core MD regions we observed related gradients of activation; for stop, for example, core activity was often strongest close to an adjacent cingulo-opercular region. Our multivariate analyses here would be sensitive to these univariate differences, in addition to pattern differences idiosyncratic to an individual participant. They produce a more complete picture of task discrimination.

Different cortical distributions of task and stimulus discrimination occurred against a background of ubiquitous discriminations significantly greater than zero. A similar result was recently reported by Xiang et al. (2024) also using HCP methods and finding widespread significant discrimination of stimulus modality. In our case, the lack of discrimination in ventricles suggest that the discrimination across all cortical parcels is real. Compared to traditional methods, the HCP protocol is known to yield more precise anatomical and functional organization of the data (Glasser et al. 2016). Results may reflect this improved data quality, along with our use of a sensitive LDC distance measure. Though the result is perhaps surprising, it is reminiscent of animal studies which, for many years, have shown the variety of neural signals observed in individual cortical areas, including motor signals in early visual areas (Mirabella et al. 2007; Niell and Scanziani, 2021), or stimulus-locked activity in motor areas (Riehle, 1991; Riehle et al. 1994). This picture is increasingly evident with recent large-scale, unselective recordings across many regions of the mouse brain (Stringer et al. 2019).

Of course, outside their effects on brain or behavior, there is no way to compare magnitudes of task and stimulus differences. To test our prediction of strong task discrimination compared to weak stimulus discrimination in MD regions, we simply chose a stimulus distinction that is very strongly signaled in brain activity. In terms of fMRI activity, faces versus buildings is one of the strongest and most widespread distinctions between visual categories, extending across large regions of occipital, temporal, parietal, and frontal cortex. This strong discrimination did not extend, however, to core MD regions. A stronger stimulus variation like modality might have shown greater discrimination in these regions.

As suggested in our previous paper (Assem et al. 2024), placement of core MD regions between regions of different cortical networks may allow selective feeding in and out of many kinds of information. Here we find that, adjacent to core MD, cortical regions discriminate both task type, and stimulus category. In a companion paper (Assem et al. 2022), we reported selective frontal lobe responses to visual versus auditory stimuli, again in regions adjacent to MD core. These discriminations in adjacent regions may be the medium by which information of different kinds arrives for its synthesis in MD core.

If core MD regions synthesize the components of a cognitive operation, why should task type be so much more strongly discriminated than stimulus category? Certainly, in the mental control program for different activities, stimulus category is only one of many components. The program for task switching, for example, must specify two stimulus-response mapping rules, the role of two colored surrounds, rules for balancing speed and accuracy, etc. All of these will be different in the n-back or stop tasks, likely contributing to the strong task discrimination we observe. That said, in our study, face versus building was not only a stimulus difference; at least in switch and stop tasks, faces and buildings were also associated with different response rules. Perhaps future work may clarify why some task elements more than others are strongly discriminated in core MD regions. Provisionally, our results suggest that higher-level, abstract features of task structure, for example the distinction between switch and stop, are more strongly coded than the lower-level component rules used within these structures. Performance differences could also contribute to between-task discrimination, for example through selective response to frequent errors in the stop task.

In a second respect, our fMRI data gave only a little support to a prediction from single unit data. In contrast to the strong nonlinearity seen in many individual neurons of monkey prefrontal cortex, we found largely linear combination of task and stimulus discrimination, with a small component of nonlinearity. This small nonlinear effect was not particularly higher in core MD regions compared to other brain networks. Of course, it is hard to move from strong nonlinearity in individual neurons to a prediction for whole fMRI vertices, each reflecting data from a very large number of neurons, each with their own pattern of selective response. Given a complex and likely nonlinear transformation of the neuronal signal, we do not have precise expectations on the magnitude of nonlinearity. Furthermore, although conjunctive coding seems essential to specify how components of a cognitive operation are combined, it would be hard to determine what level of conjunctive coding is functionally necessary. Certainly, however, we did not see strong evidence for conjunctive coding; for example, a predominance of vertices with strong face-building discrimination in one task, but not in the other (Fig. 1C, multiplicative and fully conjunctive models). The present data suggest that such conjunctive codes may be hard to detect at the parcel level, at least using current fMRI methods.

## Author contributions

Sneha Shashidhara (Conceptualization, Data curation, Formal Analysis, Investigation, Methodology, Project administration, Software, Visualization, Writing—original draft, Writing—review and editing-), Moataz Assem (Conceptualization, Data curation, Formal Analysis, Investigation, Methodology, Project administration), Matthew F. Glasser (Investigation, Methodology), John Duncan (Conceptualization, Funding acquisition, Investigation, Methodology, Project administration, Resources, Supervision, Writing—original draft, Writing—review and editing-).

## Funding

A Medical Research Council grant (SUAG/045.G101400 to J.D.); Gates Cambridge Trust (Cambridge, UK to S.S.); Cambridge Commonwealth European and International Trust (Yousef Jameel scholarship to M.A.); National Institutes of Health grant (R01MH060974 to M.F.G.).

*Conflict of interest statement*: None declared.
